# Comparative analysis on microsurgical removal of craniopharyngioma via lateral supraorbital approach and standard pterional approach

**DOI:** 10.1186/s41016-018-0126-7

**Published:** 2018-08-01

**Authors:** Chongshun Zhao, Zhouqing Chen, Na Xu, Tao Xue, Xin Wu, Wanchun You, Yun Zhu, Zhong Wang

**Affiliations:** 1 Department of Neurosurgery, The First Affiliated Hospital of Soochow University, 188 Shizi Street, Suzhou, Jiangsu Province, 215006 China; 2 State Key Laboratory of Medical Neurobiology and Institute of Brain Sciences, Fudan University, Shanghai, 200032 China

**Keywords:** Lateral supraorbital approach, Standard pterional approach, Craniopharyngioma

## Abstract

**Background:**

Craniopharyngioma is a kind of intracranial benign tumor that is primarily treated with surgery. At present, a variety of surgical approaches are used for tumor resection. We have conducted a comparative analysis of the two approaches most used in our department.

**Methods:**

The study retrospectively analyzed the clinical data from 65 patients with craniopharyngioma surgically treated by the two approaches mentioned above. Among these patients, 24 were treated by lateral supraorbital (LSO) approach and 41 by standard pterional approach. Indicators including, but not limited to, length of incision, operation time, postoperative pituitary function, urine volume, visual function improvement, and hospitalization were used to compare these two groups of patients.

**Results:**

The data shows that there was no significant difference in total tumor resection rate (*P* = 0.54), postoperative visual field improvement (*P* = 0.68) and postoperative function of endocrine. However, the LSO approach significantly reduced the operative incision (*P* = 0.001), shortened the operation time (*P* = 0.001) and operative complexity, while reducing the incidence of postoperative complications (*P* = 0.04).

**Conclusions:**

In surgical treatment of craniopharyngioma, LSO approach has similar surgical effect with standard pterional approach, but it can significantly shorten the operation time, reduce surgical trauma and the incidence of complications. Therefore, LSO provides another alternative to surgical approach for microsurgical removal of craniopharyngioma.

## Background

Craniopharyngioma is a kind of intracranial benign tumor. It has been reported that craniopharyngioma accounts for 1.2–4% of all primary intracranial tumors and 5–10% of primary brain tumors in children [1]. It originates from the third ventricle-hypothalamus -funnel-pituitary axis [2]. Based on its histological origin, craniopharyngioma often occurs in the sella region while its clinical symptoms are usually manifested as visual disturbances and endocrine disorders [3]. At present, microsurgical resection is still the first choice in the treatment of craniopharyngioma. Because of its special growth position, craniopharyngiomas are often closely attached to the hypothalamus, optic chiasm, pituitary stalk, and other important neural structures. Therefore, choosing the right surgical approach is crucial in completely removing the tumor while also protecting the patient’s vision and normal endocrine function.

The standard pterional approach has been widely used to treat craniopharyngiomas [4]. In 2005, Professor Juha Hernesniemi of the Neurosurgery Center in Helsinki, Finland, introduced the lateral supraorbital (LSO) approach as an alternative way to the possible standard pterional approach [5]. In surgical treatment of craniopharyngioma, these two surgical approaches have different advantages and disadvantages. The standard pterional approach, as one of the most widely used craniotomy approaches, has the advantages of large exposure range, clear vision, easy to grasp, etc., but also has some shortcomings such as large surgical injury and aesthetic impact of bone flaps [6]. The LSO approach provides nearly equal saddle area exposure [7] while reducing surgical trauma and simplifying the surgical procedure. Here, we conducted a retrospective study of 65 cases of craniopharyngioma in the Department of Neurosurgery, The First Affiliated Hospital of Soochow University. The results are reported below.

## Methods

### Inclusion criteria

(1) patients received plain MRI scans and enhanced scans after admission. (2) Accepted pituitary function tests before and after surgery (3) Received surgical treatment of LSO approach or standard pterional approach. (4) Postoperative pathological examination confirmed the diagnosis of craniopharyngioma. (5) Patients had no serious hematological disease or severe organ failure such as liver and kidney function failure before surgery.

### Patient population

Selected 65 patients with craniopharyngioma admitted to the Department of Neurosurgery of the First Affiliated Hospital of Soochow University from January 2011 to June 2016, among them, 56 (87.7%) were newly diagnosed and 9 (12.3%) were relapsed. All patients were divided into LSO approach group and standard pterional approach group according to the surgical approaches they accepted. Twenty-four patients (36.9%), 14 men and 10 women were treated with LSO approach. The average age was 52.0 ± 19.67 years, including 2 children (8.3%). The average longest diameter of the tumor was 3.6 ± 1.39 cm. The remaining 25 males and 16 females totaled 41 patients (63.1%) undergoing standard pterional surgery. The average age was 46.6 ± 16.19 years, including one child (2.4%). The average longest diameter of the tumor was 3.3 ± 1.09 cm. The senior author (Zhong Wang) performed 24 operations via LSO approach and 7 operations via standard pterional approach. Rest 49 operations were instead of are performed by other senior surgeons in the First Affiliated Hospital of Soochow University via standard pterional approach. Comparison of some variables in two groups of patients before operation is shown in Table 2. As is shown, there is no significant difference between two groups.

### Imaging examination

**Fig. 1:**
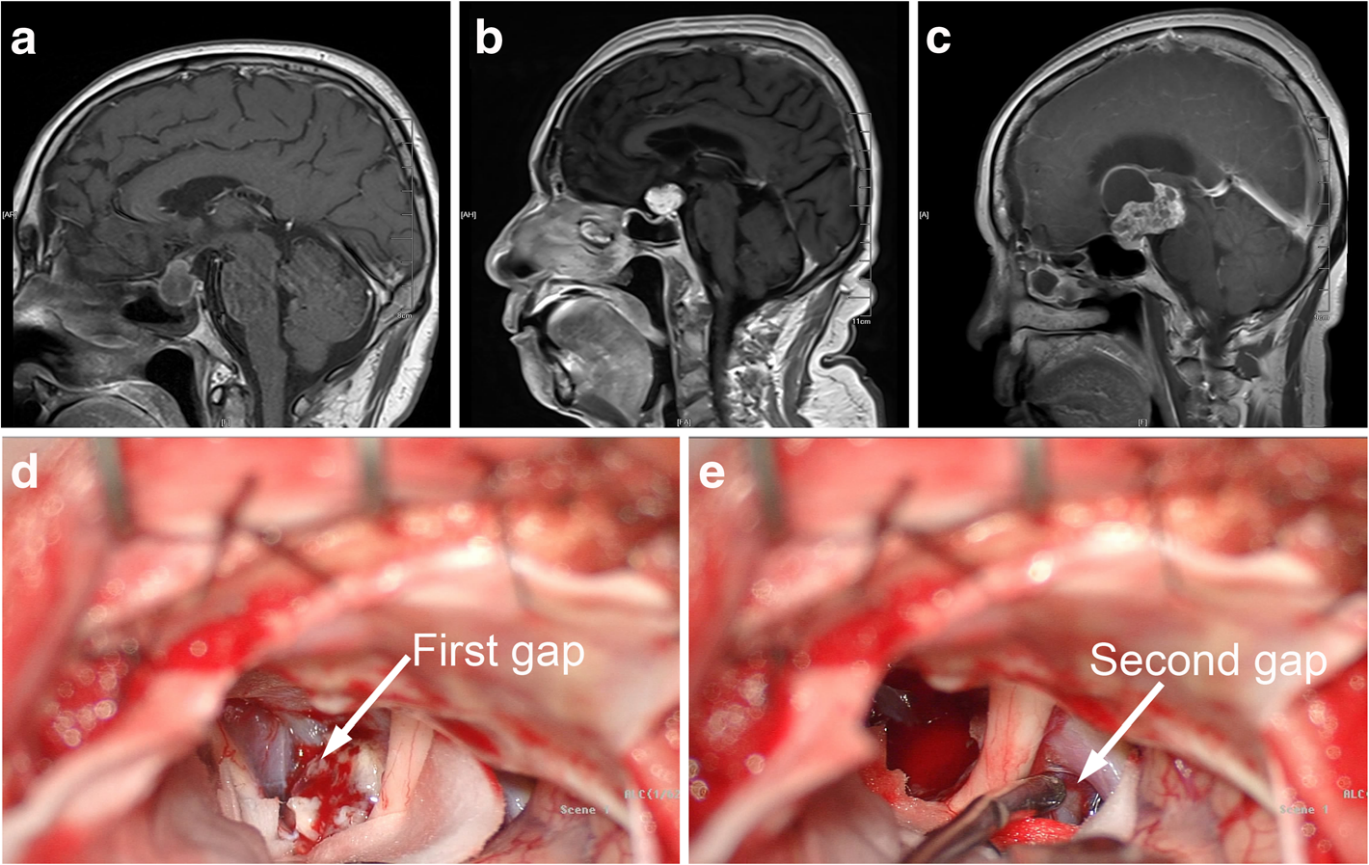
Three common craniopharyngioma MRI’s sagittal and two gaps of tumor exposure under the supraorbital visual field. **a** intra-suprasellar, **b** suprasellar-extrayentricular, **c** intra-suprasellar-intraventricular, **d** expose tumor from the first gap, **e** expose tumor from the second gap

All patients undergo CT and MRI examination before surgery. There were 14 cases (21.5%) that tumors were cystic and only reinforced with cystic wall, 42 cases (64.6%) were cystic & solid and non-uniformly enhanced, in the rest 9 cases (13.8%), tumors were solid and evenly strengthened. According to the classification method of Yasargil, tumors are divided into small (< 2 cm), medium (2-4 cm), large (4-6 cm), and huge (> 6 cm) based on the longest axial diameter of the tumor [8]. Bounded by the diaphragma sellae and the third ventricle floor, further dividing the tumors into 6 groups: intrasellar, intra-suprasellar, intra-suprasellar-intraventricular, suprasellar-extraventricular, suprasellar-intraventricular and intraventricular [9]. The distribution of tumors’ size and classification is shown in Table 1. In Fig. 1, we have exemplified three kinds of tumors. (a: Intra-suprasellar type, cystic; b: Suprasellar-extraventricular, solid and evenly strengthened; c: Intra-suprasellar-intraventricular, cystic & solid and non-uniformly enhanced).

**Table 1:** Tumor size and classification distribution.

Classification	Tumor size				
	Small	Medium	Large	Huge	Total
Intrasellar	1	0	0	0	1
Intra-suprasellar	1	18	2	0	21
Intra-suprasellar-intraventricular	1	8	4	2	15
Suprasellar-extraventricular	2	11	0	0	13
Suprasellar-intraventricular	0	10	5	0	15
Intraventricular	0	0	0	0	0
Total	5	47	11	2	65

### Endocrine examination

Fasting blood was collected from all patients before and 7 days after operation for pituitary function testing. Pituitary-thyroid hormone axis (FT3, FT4 and TSH), pituitary-adrenocortical hormone axis (Cor), pituitary-sex hormone axis (FSH and LH), and prolactin (PRL) and growth hormone (GH) were measured. After the operation, every patients’ urine output volume would be recorded by days during hospitalization. The definition of central diabetes insipidus is as follows: 24-h urine output volume exceeds 500 ml without dehydrating agent, or urine output volume per hour is greater than 250 ml (more than 6 ml/kg/h) and lasts for more than 3 h [10]. As is shown in Table 2, there is no significant difference between two groups in endocrine condition before operation.

**Table 2:** Comparison of preoperative conditions between two groups of patients

Variables	LSO approach group	Standard pterional approach group	*P* value
Classification			0.944
Intrasellar	0	1	
Intra-suprasellar	8	13	
Intra-suprasellar-intraventricular	5	10	
Suprasellar-extraventricular	5	8	
Suprasellar-intraventricular	6	9	
Intraventricular	0	0	
Tumor size (cm)	3.6 ± 1.39	3.3 ± 1.09	0.228
Sex			0.519
Female	10	16	
Male	14	25	
Age (year)	52.0 ± 19.67	46.6 ± 16.19	0.234
Pre-operation endocrine disorders			
Triiodothyronine	13	14	0.128
Thyrine	10	13	0.435
HormotHyrin	3	4	0.703
Hudrocortisone	11	22	0.612
Flitropin	8	15	1.000
Prolan B	13	23	1.000
Prolactin	17	26	0.597
Growth hormone	0	1	1.000

### Surgical technique

**Lateral supraorbital approach:** Firsttly, patients were anesthetized and gently fixed on the operating table with supine position. Then, put a surgical pillow under the patient’s shoulder to make sure that his head level slightly higher than the heart, and made the neck bent 10 ° ~ 20 ° backward. Turn the face to the contralateral side 10 ° ~ 40 ° and keep the frontal condyle at the highest point of the surgical area. Next, the incision was made at the forehead hairline terminated at the anterior arch of the ear and the frontal cristae should be exposed well. Then, pulled down the musculocutaneous flap and cut a small part of the anterior temporalis muscle. A hole was made near the ipsilateral frontal condyle by drill and cut out more bone flaps to enlarge the bone window (diameter 3~ 4 cm) with milling cutter. In order to expose basis cranii interna well, a burr could be used to remove the extra bone if necessary. Continually, suspend and cut the dura, push the frontal lobe with brain spatula and released cerebrospinal fluid by opening the internal carotid artery pool and optic cross pool. As is shown in Fig. 1(d & e), the tumor was exposed the through the first gap and second gap and the adjoining relationship between the tumor and adjacent tissues were observed. If the optic nerve of the patient is short or there exist other factors affect the exposure of the tumor, the front of the sylvian fissure should be opened for more space.

**Standard pterional approach:** Firstly, patients were anesthetized and gently fixed on the operating table with supine position. According to the preoperative images, put a surgical pillow under the patient’s shoulder to make sure that his head level slightly higher than the heart, and made the neck bent ~ 10° backward. Turn the face to the contralateral side 30°~ 45° and keep the frontal condyle at the highest point of the surgical area. Cut the skin at 1 cm in front of the tragus above the zygomatic arch and then made the coronal incision at the hairline, which end at approximately 2 cm away from the midline. The fully layer of skin flap was separated. After that, pulled down the temporalis muscle to the zygomatic arch nearby the frontal condyle. The first hole was drilled between frontal zygomatic suture and tarsal line. The second hole was located at 1~ 2 cm above the supraorbital foramen and the third hole was on the parietal bone. The last hole was made 4 cm below the third hole and 3 cm behind the first hole. After that, removed the bone flap with a milling cutter. By using rongeur and drill, the lateral side of the sphenoid iliac crest was abraded. Suspended and cut the dura, then properly separated the lateral fissures and released cerebrospinal fluid under the microscope. Gently retracted the frontal lobe and exposed the tumor.

### Statistical analyses

We use SPSS software version 23.0 to perform the statistical analysis. The kolmogorov-Smirnov test is used to test the normality of quantitative data distribution. Continuous variables are expressed as means and categorical variables are expressed as percentages. Analysis of continuous variables using ANOVA test (parameter data) and Mann-Whitney U test (nonparametric data), while the analysis of categorical variables using Chi-square test or Fisher’s exact test. A *P* value< 0.05 was considered as significant difference.

## Results

### Surgical results

The operation time of the LSO approach group was 205.4 ± 65.71 min, which was significantly shorter than the standard pterional approach group of 289.9 ± 89.89 h (*P* = 0.001). Furthermore, the incision length in the LSO approach group was 8.21 ± 1.91 cm, significantly shorter than the standard pterional approach group, which is 15.07 ± 3.47 cm (*P* = 0.001). According to surgery video and postoperative enhanced MRI examination, 20 (83.3%) cases were totally resected and 4 (16.7%) cases were partially removed in the LSO approach group. In the standard pterional approach group, 35 (85.4%) cases were completely resected and 6 (14.6%) cases were partially resected. After Fisher’s exact test, there was no significant difference between the LSO approach group and the standard pterional approach group in whether the tumor totally cut (*P* = 0.54) (Table 3).

**Table 3:** Surgical results

	LSO approach	Standard pterional approach	*P* Value
Operation time (Min)	205.4 ± 65.71	289.9 ± 89.89	0.001
Incision length (Cm)	8.21 ± 1.91	15.07 ± 3.47	0.001
Tumor resection			0.545
complete resection	20(83.3%)	35(85.4%)	
partial resection	4(16.7%)	6(14.6%)	
Hospitalization days (Day)	22.25 ± 7.89	24.80 ± 11.68	0.258
Postoperative hospital days (Day)	16.41 ± 7.42	18.66 ± 11.67	0.220

### Postoperative endocrine conditions

**Fig. 2:**
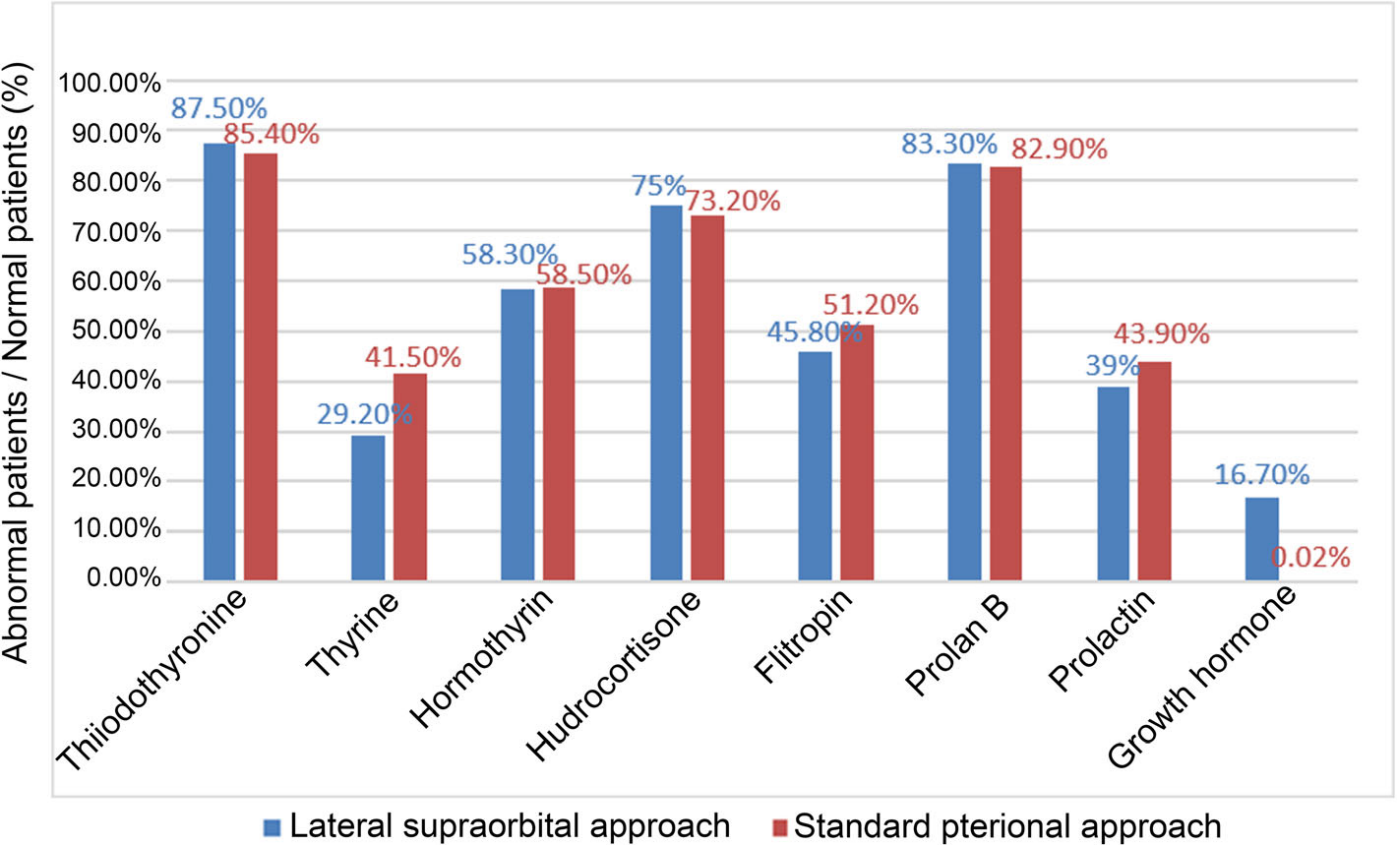
Contrast LSO approach and Standard pterional approach postoperative hormone secretion dysfunction. The LSO is represented by blue column and the standard pterional approach is represented by red column

**Fig. 3:**
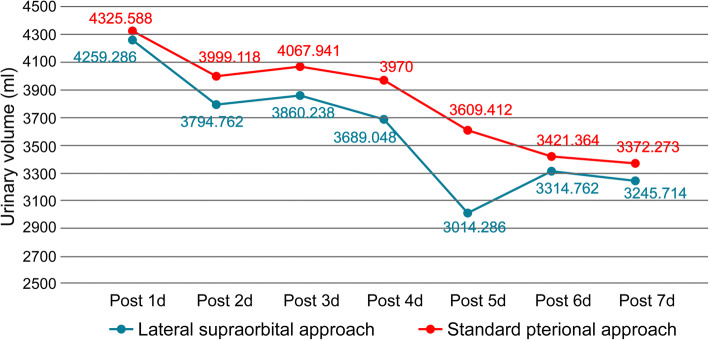
Contrast LSO approach and Standard pterional approach average daily urine volume within a week after surgery. The LSO is represented by a blue line, and the standard pterion path is indicated by a red line

Seventh days after the operation, all patients had 1 or more hormone secretion dysfunction after operation. The specific statistics are shown in Fig. 2. Diabetes insipidus was found in 14 (58.3%) patients in the LSO approach group and 30 (73.1%) patients in the standard pterional approach group. According to statistical analysis, there was no significant difference in the proportion of patients with diabetes insipidus after the treatment of craniopharyngioma between the LSO approach group and the standard pterional approach group (*P* = 0.22). The average daily urine volume within a week after surgery is shown in Fig. 3.

### Postoperative visual acuity

After the operation, we continuously monitor the patient’s visual status until discharged. The visual status of the LSO approach group was obviously improved in 13 (54.2%) cases while the number in standard pterional approach group is 20 (48.8%). There was no significant difference between the two groups. (*P* = 0.68).

### Postoperative hospitalization

There were 2 (8.3%) cases which had postoperative complications after operation in the LSO approach group. One case was subdural effusion and another was pulmonary infection. Both of them were cured and discharged after treatment. In the standard pterional approach group, postoperative complications occurred in 12 (29.3%) cases, including 1 case of hydrocephalus, 3 cases of pulmonary infection, 2 cases of central hyperthermia, 2 cases of wound infection, 1 case of wound infection with central nervous system infection and 2 cases of severe endocrine dysfunction, and these 11 cases were cured or obviously improved after treatment. One case died due to severe electrolyte imbalance with pulmonary infection and massive bilateral pleural effusions. There was a statistically significant difference in postoperative complications between the LSO approach group and the standard pterional approach (*P* = 0.04). The mean length of hospital stay in the LSO approach group was 22.25 ± 7.89 days, and the mean length of hospital stay after operation was 16.41 ± 7.42 days after surgery, which was shorter than the standard pterional approach group of 24.80 ± 11.68 days and 18.66 ± 11.67 days, but was not statistically significant (Table 3).

## Discussion

A number of advances have been made in the treatment of craniopharyngioma in recent years, such as transnasal endoscopic approach, keyhole approach [11], intratumoral injection of bleomycin [12] and intraluminal radiotherapy [13] etc. Currently, a large number of literature about transnasal endoscopic approach for craniopharyngiomas suggest that the use of endoscopy can achieve equal effects and less surgical damage than craniotomy [14]. The main disadvantage is the higher incidence of cerebrospinal fluid leakage and the requirements for the surgical instruments, yet many neurosurgeons still perfer craniotomy. As currently reported, total resection rate of craniopharyngiomas has reached more than 90%, while the mortality rate is less than 5% [15]. The choice of best surgical approach is still under discussion as a result of change in location of the craniopharyngioma and biases of various neurosurgeons regarding the different approaches. Common approaches include: standard pterional approach, frontal approach, transsphenoidal approach, translongitudinal-septal approach, and transfrontal-anterior longitudinal split-end plate approach approaches. These approaches get in to the saddle area from the side, front, lower front, and upper to expose the tumor. Each surgical approach has its advantages and disadvantages. Since the pterion approach in the 1990s, advocated by Yasargil, the standard pterional approach has been known as the most commonly used surgical approach for craniopharyngioma for quite a long time. It is generally confirmed that the standard pterional approach is the shortest way to reach the saddle area, meanwhile, separating ipsilateral sylvian fissure, which provide more surgical space and easier for surgeons to do operations. However, for the treatment of craniopharyngioma, the disadvantages of standard pterional approach cannot be ignored. Entering the saddle area from the side will inevitably bring about difficulties in revealing the contralateral structure. The medial side of the optic nerve will often become the blind area of the operative field and become a high incidence of tumor residue. In contrast, the LSO approach can easily obtain the surgical field by using the corridor between two optic nerves. In fact, as an alternative approach to the standard pterional approach, the LSO approach has many different features. Combined with relevant literature and our experience in surgery, we would like to discuss some different operational skills in these two approaches.

At the same time, the standard pterional approach will cause greater damage to patient’s lateral malleolus, and often damage the facial nerves of the patient, resulting in postoperative chewing dysfunction, facial aesthetics and other side effects. It was reported that separating the skin flap between the subgaleal and temporalis muscle would result in a 30% incidence of facial nerves injury [16, 17]. Separating the skin flap directly from the muscle and skull, and then turning over the skin and temporalis muscle at the same time will better protect the nerve but will reduce the surgical field near the skull base. Many efforts have been made to protect the superficial temporal artery and the facial nerve in standard pterional approach. But the extensive exposure and separation of the temporalis are inevitable. As an alternative choice of standard pterional approach, the LSO approach has its unique features and advantages. In the craniotomy of the LSO approach, only a small part of the temporalis muscles is cut, which may reduce the possibility of patients with temporalis atrophy or chewing dysfunction and other sequelae. Traditionally, facial nerve rarely injured in the LSO approach, but according to the relevant reports, the frontal branch of the facial nerve can be damaged if the incision beyond the lateral of zygomatic process is too much, causing temporary or permanent disappearance of forehead wrinkles. Another possible of LSO approach is the injury of the supraorbital nerve. In this study, the operative time and surgical incision length of the LSO approach group were significantly shorter than the standard pterional approach group, demonstrating that the LSO approach can simplify the surgeon’s operation and reduce the patient’s surgery trauma. Besides, none of the patients treated by LSO approach in this study was placed with an epidural drainage tube, which reduced the probability of encountering a problem in the removal of the drainage tube, cerebrospinal fluid leakage or intracranial infection.

Due to the growth manner and growth pattern of craniopharyngioma it is inevitable that the tumors involve the pituitary stalk, optic nerve, and hypothalamus. Although fine-grained operations under the microscope and sharp separation of key parts are commonly used to protect the surrounding tissues, complications such as pituitary dysfunction, central diabetes insipidus, visual disturbance or hypothalamic symptoms are still common after craniopharyngioma surgery. It has been reported that the incidence of diabetes insipidus after total excision of craniopharyngioma can reach 79% [17]. At the same time, there are documents showing that the complete extent of craniopharyngioma resection is positively correlated with the occurrence rate of diabetes insipidus [18]. In this study, the incidence of postoperative diabetes insipidus was 67.7%. There was no statistical significance in the comparison between the two surgical approaches. Eleven (16.9%) cases have symptoms of diabetes insipidus before surgery, and all of them had diabetes insipidus after the operation. Paja [16] reported that none of the patients who have preoperative pituitary hormones disfunction had improved after surgery while most or all of the other patients had hypopituitarism after surgery. In this study, all patients had abnormal secretion of one or more hormones after surgical treatment. But there was no significant difference in the endocrine dysfunction between the two surgical approaches.

It must be admitted that there are many deficiencies in our research. First of all, the choice of surgical approach in this research is not random or following a fixed criteria. It is determined by the size, location of the tumor, the patient’s condition, and other factors while also influenced by the surgeon’s personal experience. Secondly, some variables need to be quantified such as the injury of the facial nerve, the improvement of visual status and the Influence of different surgeons on operation process and results. However, with appropriate research design and data statistics, we believe that our conclusion is reasonable and meaningful.

## Conclusion

In summary, for the surgical treatment of craniopharyngioma, the LSO approach and the standard pterional approach have no significant difference in the total tumor removal rate, protection of endocrine function, and improvement of visual function. However, LSO approach can significantly shorten the surgical incision, reduce the operation time and operation complexity, and lower the incidence of postoperative complications, which explains why the LSO approach can be a qualified alternative to the standard pterional approach and should be considered in the treatment of craniopharyngioma. However, this study has some deficits such as insufficient of sample sizes, lack of analysis of pathological types, etc. There is still no consensus on the location classification of craniopharyngiomas and the best surgical approach for different types of craniopharyngioma still remains to be further researched.

## Abbreviations

Cor: Cortisol hormone
CT: Computed tomography
FSH: Follicle-stimulating hormone
FT3: Free triiodothyronine 3
FT4: Free triiodothyronine 4
GH: Growth hormone
LH: Luteinizing hormone
LSO: Lateral supraorbital
MRI: Magnetic resonance imaging
PRL: Prolactin
TSH: Thyrotrophic stimulation hormone
